# Type of bilingualism conditions individual differences in the oscillatory dynamics of inhibitory control

**DOI:** 10.3389/fnhum.2022.910910

**Published:** 2022-07-28

**Authors:** Sergio Miguel Pereira Soares, Yanina Prystauka, Vincent DeLuca, Jason Rothman

**Affiliations:** ^1^Department of Linguistics, University of Konstanz, Konstanz, Germany; ^2^Language Development Department, Max Planck Institute for Psycholinguistics, Nijmegen, Netherlands; ^3^Department of Language and Culture, UiT the Arctic University of Norway, Tromsø, Norway; ^4^Nebrija Research Center in Cognition, University of Nebrija, Madrid, Spain

**Keywords:** bi-/multilingualism, cognitive control, time-frequency representations (TFRs), brain oscillations, Flanker task

## Abstract

The present study uses EEG time-frequency representations (TFRs) with a Flanker task to investigate if and how individual differences in bilingual language experience modulate neurocognitive outcomes (oscillatory dynamics) in two bilingual group types: late bilinguals (L2 learners) and early bilinguals (heritage speakers—HSs). TFRs were computed for both incongruent and congruent trials. The difference between the two (Flanker effect vis-à-vis cognitive interference) was then (1) compared between the HSs and the L2 learners, (2) modeled as a function of individual differences with bilingual experience within each group separately and (3) probed for its potential (a)symmetry between brain and behavioral data. We found no differences at the behavioral and neural levels for the between-groups comparisons. However, oscillatory dynamics (mainly theta increase and alpha suppression) of inhibition and cognitive control were found to be modulated by individual differences in bilingual language experience, albeit distinctly within each bilingual group. While the results indicate adaptations toward differential brain recruitment in line with bilingual language experience variation overall, this does not manifest uniformly. Rather, earlier versus later onset to bilingualism—the bilingual type—seems to constitute an independent qualifier to how individual differences play out.

## Introduction

Attaining competencies in and managing more than one language in a single mind is complex and dynamic. Because all languages in the mind maintain some level of activation, irrespective of apparent contextual need, there is a ubiquitous demand to manage them (*via* suppression and/or selection, Kroll et al., 2012). This mental juggling is argued to lead to adaptations in domain-general control, where cognitive and language control networks overlap (see e.g., Anderson et al., 2018a). While effects are not always replicated (see Lehtonen et al., 2018), studies have shown that, at least under certain conditions, the brain adapts structurally, functionally and chemically to bilingual experience (e.g., Stocco et al., 2014; Abutalebi and Green, 2016; Weekes et al., 2018; DeLuca et al., 2019a; Pliatsikas, 2019; Grundy, 2020; Pliatsikas et al., 2021). And yet, the study of bilingualism and neurocognition has primarily focused on monolingual vs. bilingual (dichotomous) group comparisons across a variety of domains and tasks (see Salig et al., 2021 for review). While such an approach has led to keen insights into the bilingual mind and brain, it has also resulted in the nature of individual-level variables across bilinguals themselves to not be adequately addressed (Pliatsikas et al., 2020; Salig et al., 2021).

Over the past few years in particular, theoretical and empirical work has hypothesized and shown the usefulness of measuring and treating dual (multiple) language experiences as continuous variables (e.g., Luk and Bialystok, 2013; Li et al., 2014; Bialystok, 2017; Grundy et al., 2017; DeLuca et al., 2019b, 2020; Surrain and Luk, 2019; Gullifer and Titone, 2020; Di Pisa and Rothman, 2021; Marian and Hayakawa, 2021). In the real world, the shapes and forms with which exposure, experience and engagement with multiple languages dynamically present themselves over individual lifespans are nearly limitless. Age (of onset and time of testing), context (in and out of community immersion), linguistic proficiency and other seemingly categorizing proxies, while important factors with considerable explanatory coverage, are not the only variables that differentiate aggregates of bilinguals nor the individuals that comprise them (e.g., simultaneous vs. sequential bilinguals, early versus later child/adult bilinguals, special populations like translators and interpreters). Rather, variation in key factors falling within specific language history backgrounds across space and time (quantity and quality of input, intensity of exposure, patterns of language use, language switching, fluctuating dominance, language community size, linguistic social networks and more) delimit individual opportunities for linguistic bilingual engagement. Their equivalence and/or their potential impact on the outcomes we aim to measure cannot be taken for granted (Leivada et al., 2021).

Acknowledging and dealing empirically with the above reality has manifold consequences. Indeed, several recent models make distinct predictions regarding specific effects for duration and extent of engagement with bilingual experience (Stocco et al., 2014; Abutalebi and Green, 2016; Grundy et al., 2017; DeLuca et al., 2020; Pliatsikas, 2020). In this light, it seems reasonable to ponder the extent to which some of the discrepancies within the empirical record might be better explained in relation to the (non-)comparability of how important individual-level variables are distributed across participants (Leivada et al., 2021). If so, it could be simultaneously true that bilingualism affords no effects to the mind/brain under some conditions (Paap et al., 2015) while translating into considerable ones (along a continuum) given distinct individual experience profiles. The overarching question and focus of research, thus, shifts to unpacking the constellations of experiences with dual (multiple) languages that give rise to effects (Grundy, 2020). However, if individual patterns of dual language engagement matter, it cannot be assumed that such patterns align symmetrically such that bilingual type (macro) categorizing factors do not intercede to affect how individual bilingual engagement patterns ultimately manifest. It is with this in mind that the present study is couched and contributory. Specifically, treating bilingualism as a continuous variable, we regress individual measures of bilingual engagement to understand how they might predict on-task neural dynamics while performing a Flanker task. However, bringing together two distinct types of bilinguals–early native (heritage speakers) and late(r) L2 learners–we further ask whether the age of onset of bilingualism interacts differentially with how proxies of bilingual engagement present in individual differences. Foreshadowing our results, while it is the case that individual patterns of dual language engagement predict individual differences in on-task TFRs in both bilingual type groups, the patterns are distinct within each group, suggesting that onset timing and/or overall duration of bilingualism has a moderating effect for how individual differences unfold.

Regarding the potential for experience related brain adaptations, heritage speaker bilingualism provides a unique and understudied test case. Heritage speakers (HSs) are early bilingual native speakers of a minority language (the heritage language) who grow up in a majority language context (Rothman, 2009; Montrul, 2016; Polinsky, 2018). HSs are typically, although not exclusively, studied and described in an adult state of linguistic knowledge (Kupisch and Rothman, 2018). Although degree of linguistic proficiency in a heritage language at the individual level varies considerably, partially overlapping with the outcome continuum reported for late(r) second language (L2) acquirers, the degree and contexts of exposure, age of acquisition, patterns of use and language switching, among many other factors definitively distinguish them. In both cases, however, experiential factors are deterministic for individual linguistic variation (Polinsky and Scontras, 2020). And yet, few studies in the neurocognition of bilingualism have looked specifically at HSs (or at least labeled and differentiated them as such).

Under the hypothesis that individual level engagement with bilingual language experience is ultimately the (most) deterministic factor for (degree of) bilingualism-induced domain-general neurocognitive effects, it is not clear if age-of-acquisition (AoA) should matter. Under what we refer to as the strong version of this hypothesis, AoA would not bring anything independent to bear, at least per se. In other words, there would be no implied potential for a maturational effect on how the brain deals with the mental exercise induced by managing more than one linguistic system. Ultimately, under such an approach, quantity and quality of engagement, if matched across subjects of different bilingual types, would yield no differences, making them the singular driving forces behind adaptation at the individual level independent of AoA. Alternatively, under what we refer to as the weaker version of this hypothesis, AoA could matter for two partially exclusive reasons: (i) there is some type of maturational effect on how the brain adapts to the same quantity and quality of dual language experience or (ii) there is no maturational effect, but type of bilingualism entails greater and lesser likelihood for intensity of dual language experience itself. This would be the case, if, on average, HSs are more likely to have deeper experience with dual language management than later onset bilinguals. That is, earlier bilinguals might be more likely to have more opportunities for experiences that translate into increased neurocognitive adaptations. For example, on average, HSs might be much more likely to engage (deeply) in activities such as code-switching that are argued to be especially relevant for degree of neurocognitive effects (Green and Wei, 2014; Hofweber et al., 2020). And so, controlling for time of bilingualism—comparing 20-year-old HSs who have been bilingual for 20 years to 35-year-old L2 learner who also have been bilingual for 20 years—does not necessarily entail that AoA alone teases out the relevant factor of potential distinction precisely because over the same time period one or the other type of bilingual might be much more likely to have deeper dual language experience. As a result of either scenario of the weaker hypothesis, HSs could show different patterns as compared to L2 learners but for quite distinct reasons. Given what we now understand as the highly neuroplastic nature of the human brain over the lifespan (Fuchs and Flügge, 2014), we take the null hypothesis to be that there is no maturational effect on how the mind/brain will adapt to the same quantity and quality of dual language experience. However, we leave open the extent to which different bilingual types will, in their aggregates and indeed across the individuals that comprise them, differ in terms of empirical exponents of cognitive tasks likened to bilingualism-induced adaptations. Why? Precisely because it is highly likely that in the most common cases bilingual type will matter for quantity and quality of opportunities to engage (and thus change) the relevant underlying cognitive systems.

Despite the above provisos, it is likely, however, that HSs have been included in the aggregate young adult bilingual groups in published neurocognitive studies, collapsed with other types of non-native bilinguals. This is potentially problematic to the extent that age-of-acquisition (AoA) could itself be deterministic above and beyond individual differences with bilingual experiences or serve as a qualifier for how they manifest. By separating these two types of bilinguals, with sufficient numbers in each group, our aim is to directly address this possibility. HSs are not only more likely to have younger AoA, but their exposure to and engagement with the two languages are likely to be on average qualitatively distinct from other bilingual types while also showing a greater range of within group variation (Pascual y Cabo and Rothman, 2012; Kupisch and Rothman, 2018). HSs in the European context at least, where English is ubiquitously taught at early ages and for which populations tend to have good competence when tested in young adulthood, are often multilinguals in the relevant sense in higher proportions than sequential L2 bilinguals. Let us imagine a potential scenario in the German context to understand why this should matter. A study might endeavor to examine the effects of bilingualism on property/domain X with young adult German L1 to English as an L2. If German monolingual learners of L2 English are combined with heritage speakers of Turkish—of which there are millions—also native L1 speakers and dominant in German to form the so-called bilingual group, deterministic variables that make them distinct are being overlooked or ignored. This reality, which we surmise is more common in practice than one might expect, would render relevant combined groupings an amalgamation of bilinguals and multilinguals. We do not (yet) know what differences, if any, juggling three or more languages confers above and beyond two. Moreover, the contexts and frequencies of how languages distribute in these bilinguals and multilinguals are not only likely to be quantitatively, but also qualitatively distinct. Collapsing them might be adding noise to the proverbial signal research endeavors to isolate. The present study, thus, takes these above provisos most seriously. This is not only one of the first studies to capitalize on and separate out HSs as a distinct group in the neurocognitive study of bilingualism, but we also examine HSs alongside a separate group of true additive L2 bilinguals. In our view, such an approach is not only beneficial to address our primary research questions, it is also in line with calls in bilingualism literatures for alternative group contrasts to sidestep the potential for a monolingual-to-bilingual comparative fallacy (e.g. Ortega, 2013; DeLuca et al., 2019b). Doing so enables us to investigate the relevant contribution and weighting of experience related variables specific to bilingualism and multilingualism and macro group level factors such as AoA, duration of being bilingual/multilingual and exposure/usage patterns in isolation as well as how they might interact with each other.

As mentioned above, bilingual experience has been shown to affect domain general cognitive control processes. The Flanker task, which we use herein, is a commonly used task to examine cognitive control including studies examining effects of bilingualism and the neural underpinnings thereof (see e.g., Van den Noort et al., 2019). However, to our knowledge, very few studies to date have examined the effects of bilingualism on the neural oscillatory dynamics related to inhibitory control (e.g., Calvo and Bialystok, 2021). Differently from the more commonly used EEG method of event related potentials (ERP) (see for reviews Grundy et al., 2017; Cespón and Carreiras, 2020), the analysis of time-frequency representations (TFR) decomposes the EEG signal into the frequency domain. This can be done at the pre-stimulus time window, as well as post-stimulus time of interest. This oscillatory activity is generated from groups of neurons that synchronize and fire together tuned to internal and external stimuli (Engel et al., 2001; Buzsaki, 2006; Cohen, 2017). Oscillations are quantified by measuring frequency-specific power from the EEG/MEG signal (Varela et al., 2001). In humans, five frequency bands have been defined based on their initial clinical relevance: delta (1–2 Hz), theta (3–7 Hz), alpha (8–12 Hz), beta (13–30 Hz), and gamma (30–200 Hz) (Buzsaki, 2006). Decades of research in cognitive neuroscience have highlighted the role of rhythmic periodic brain activity in coordinating large-scale cortical networks to sustain cognitive processing and enable humans to pursue goal-directed behavior (Thut et al., 2012). Changes in environmental conditions need to be efficiently picked up by the executive functions system in order to allocate more attention and cognitive resources to a selected task, while at the same time being able to suppress distracting information (Botvinick et al., 2004). Conflict management and suppression of task-irrelevant information are typically related to top-down executive control mechanisms in the prefrontal cortex (PFC), particularly in the anterior cingulate cortex (ACC) (Botvinick et al., 2001; Fan et al., 2003; Helfrich and Knight, 2016; Haciahmet et al., 2021).

Consistently, EEG and magnetoencephalography (MEG) studies have linked power changes in prefrontal theta activity to temporal reorganization of neural networks coinciding with decision points, i.e., action monitoring and selection (Cavanagh et al., 2012). Similarly, a considerable amount of work has found midfrontal theta power and phase synchronization in frontal electrode clusters (ACC and PFC) associated with stimulus conflict detection and response monitoring (Pastötter et al., 2013; Cavanagh and Frank, 2014; Oehrn et al., 2014; Duprez et al., 2018; Brunetti et al., 2019; Pscherer et al., 2021). Although frontal theta appears to be the optimal candidate for regulating and modulating executive control functions, it is not the only one. Current research has demonstrated how the collective participation of multiple frequency bands (hence several underlying cognitive regulating phenomena) more reliably underpins preparation and implementation of cognitive control (Cooper et al., 2016). Specifically, delta has been found to regulate rule implementation and alpha motor response and anticipatory updating mechanisms (Cooper et al., 2016). Alpha has also been associated with the regulation of pre-stimulus proactive control mediated by the superior frontal cortex (SFC) (Freunberger et al., 2008; Suzuki et al., 2018). Furthermore, increases in alpha power are a proxy for the gating of contributions from task-irrelevant cortical areas (Jensen and Mazaheri, 2010; Jensen et al., 2012). Finally, beta has been associated with several higher cognitive processes, among others top-down selective attention (Siegel et al., 2011).

Studies in bilingual neurocognitive adaptations so far have mostly been based on functional magnetic resonance imaging ((f)MRI). Generally, dual language use leads to brain changes both anatomically and functionally (Del Maschio and Abutalebi, 2019; Pliatsikas, 2019), especially in older populations (Bialystok et al., 2012; Gallo et al., 2020). Furthermore, individual language experiences differentially affect these brain adaptations (DeLuca et al., 2019b; Pliatsikas et al., 2020; Gallo et al., 2021). Related literature using EEG is limited and (almost) exclusively looks into related ERPs signatures (see Grundy et al., 2017; Cespón and Carreiras, 2020 for review). The strength and advantage of adopting EEG over (f)MRI is that it accommodates for the investigation of rich neural processing at the millisecond resolution level and can provide crucial information on both strength and timing of cognitive processes (Luck and Kappenman, 2011). In complement to ERPs, TFR is a welcome approach because it allows the capturing of multiple simultaneously occurring cognitive processes (Bastiaansen et al., 2011; Prystauka and Lewis, 2019).

While the use of oscillations to examine the neural underpinnings of language comprehension and sentence processing is increasing (see Prystauka and Lewis, 2019 for a review), this method has not been widely applied to look at potential bilingualism-induced effects on domain-general cognition. A series of resting-state EEG (rs-EEG) (a measure of the ongoing brain signal in a task-free context) studies have investigated immersive computerized (second) language learning paradigms (Prat et al., 2016, 2019). Results reveal correlations between learning outcomes and resting-state low-, mid- and high-beta power (Prat et al., 2016) and functional connectivity across all frequencies correlating with posttest memory retention and speech variance during learning (Prat et al., 2019). Although these two studies are done in the context of bilingual language learning, they highlight how oscillatory dynamics can be profitably employed in bilingualism research more generally to better understand underlying processes of neural computation serving language related functions.

Bice et al. (2020) were the first to apply neural oscillations with the intention to investigate if underlying functional brain adaptations can be predicted by bilingual experiences. Comparing rs-EEG data, they found that bilinguals exhibited higher alpha power and coherence in the alpha and beta frequency ranges over monolinguals, positively correlating with language background measures. Similarly, Pereira Soares et al. (2021) correlated rs-EEG with bilingual experience measures to investigate if and how determinants of bilingualism reshape the mind/brain. The findings revealed modulatory effects of age of second language acquisition on high beta and gamma power, whereas higher degree of use of the second language at home and in society contexts correlated with functional connectivity (mean coherence) in theta, alpha and gamma frequencies. In summary, the results of these two studies highlight the modulatory role of brain oscillations (measured in a task free context) in dual language scenarios and underline how these effects vary by degree of individual engagement with bilingualism experience factors over the lifespan.

The present study tests three hypotheses guided by the recent Unified Bilingual Experience Trajectories (UBET; DeLuca et al., 2020) framework. UBET makes detailed predictions about how four general components of bilingual experience (intensity/diversity of use, language switching, relative proficiency, and duration of use) would variably drive adaptations in cognitive control and its neural underpinnings. As pertains specifically to adaptations in oscillatory dynamics, several of these predictions from the UBET framework are key: increased intensity and diversity of bilingual experience would positively correlate to theta power (in situations of cognitive control). This theta increase is predicted to stem from increased fronto-cortical recruitment to handle the higher control demands associated with this experience. Alternatively, prolonged duration of bilingual experience would relate to increased alpha suppression/desynchronization in situations requiring cognitive control, and decreased theta activity. This shift in oscillatory dynamics is related to a transition toward increased efficiency and automation of handling these existing control demands. Crucially, intensity of use would have an additive effect in this transition, that is, increased intensity of exposure would modulate the latency by which adaptations to efficiency manifest neurophysiologically. Accordingly, our predictions were as follows:

(1) *Bilingual group types differences*: power differences at the group level between HSs and L2 learners, specifically increased theta activation for L2 learners and increased alpha suppression for HSs for interference suppression given the difference in duration commensurable with bilingual type.
(2) *Individual differences predicted by engagement patterns*: collapsing the groups and regressing dual language engagement (i.e., how much and in what contexts they use both languages) as continuous variables, one might find correlations between power in theta (synchronization reflected in frontal electrode clusters) reflecting functional adaptations to increased control demands and alpha (suppression) with engagement above and beyond bilingual type specific effects, reflecting increased efficiency in handling control demands
  and/or
  If intensity over duration of engagement has an additive effect, we might expect different patterns of individual differences between HSs and L2 learners given inherent differences regarding language usage distribution. For example, one could expect all HSs to use a non-societal language at home (potentially the only context in which they use their heritage language). Thus, high use at home would not necessarily signal overall intensity at an equivalent level an equal score would for an L2 learner. In the latter case only, since the non-societal language is not expected in that context, a high score in home use is likely to denote a rather high level of intensity overall.
(3) *Correspondence between neural dynamics and behavioral performance*: theta activation is predicted to correlate with faster reaction times in the Flanker paradigm. Alpha suppression is predicted to show a greater dissociation with reaction times, especially with prolonged duration of bilingual engagement, signifying increased efficiency of handling control demands.

## Materials and methods

### Participants

Data were collected from 60 bi-/multilingual participants (43 female), of which 32 were L2 learners outside of immersion (English in Germany) and 28 were early bilinguals (heritage speakers of Italian in Germany). The L2 learners spoke German as L1 and English as L2, whereas the HSs had Italian as their L1 and either acquired German simultaneously as their second L1 (2L1) or had acquired German from a very young age in Germany, below 4 (Meisel, 2011). The age that participants were exposed for the first time to bilingualism (the L2/2L1) (mean AoA for L2 = 9.4y; SD = 1.98y, mean AoA for 2L1 = 1.88y; SD = 1.7y) and their crucially contexts of bilingualism (immersion or not) differed, but age at time of testing did not (mean age for L2 = 24.65y; SD = 3.59y, mean age for 2L1 = 24.57y; SD = 3.41y). Although the majority of our participants were first exposed to the other language(s) at a young age, this does not exclude that meaningful variation afforded by the context of each individual's bilingual language use is washed out. Given the status of the L2/L3 (English) as *lingua franca*, timing of first exposure can be misleading, i.e., quantity, quality and intensity of exposure and use can vary even at such an early age especially outside of a native English immersion context. On the other hand, heritage bilingualism is, by definition, naturalistic. Provided that the home language is in active use in its domains, heritage bilingualism places, at least at young ages, individuals in a context of immersive opportunities with ample exposure and diversity of active use of the two languages. Especially over development/maintenance through young adulthood, however, it presents a plethora of mitigating circumstances for interindividual variation in linguistic proficiency outcomes and language engagement (e.g., Kupisch and Rothman, 2018; Polinsky, 2018). Notwithstanding differences from monolingual baselines, it is generally accepted that HS grammars are not only native and naturalistic, but also comprehensive, coherent, and universally compliant with natural language (Pascual y Cabo and Rothman, 2012; Rothman and Treffers-Daller, 2014; Lohndal et al., 2019; Polinsky and Scontras, 2020). And so, regardless of the reported early first exposure to English of the L2 group, there are considerable and important differences to the “earliness” of HSs linguistic onset and temporal exposure/engagement to bilingualism.

Since all participants are sampled from the same context in Germany where English is taught pervasively and early on, the heritage speakers have also been exposed to English, making them multilinguals. Their proficiency in English, in fact, did not differ from that of the L2 group. This is unsurprising since their trajectories with English over time are not expected to be, and were indeed not, different in the aggregate from the L2 learners in our sample. Thus, while all are at least bilingual, what distinguishes our groups is their native bilingualism with Italian (or not). In addition to more fine-grained measurements of linguistic exposure and engagement, described separately below, Socio-Economic Status (SES) was coded, from 0 to 4, based on the participant's mother's highest level of education (0 = lower than a high school diploma, 4 = postgraduate degree). The mean SES was 1.15 (SD = 1.22), and there were no differences across the groups (*t*(55) = 1.99, *p* = 0.052).

### Background measures

Participants completed the Language and Social Background Questionnaire (LSBQ) (Anderson et al., 2018b), which documents language exposure and use throughout the lifetime in a wide range of settings and activities. The LSBQ factor calculator provides three different (weighted) composite scores derived from a subset of relevant questions: language use in the home environment (Home), language use in social contexts (Social), and language proficiency in the societal majority language (Proficiency). Regarding both Home and Social factors, the higher the score is, the greater the engagement in the non-societal language is. Alternatively, a lower score indicates more use and exposure with the societal language in a given context. As for Proficiency, higher scores reflect increased proficiency in the societal majority language (in our case German). Additionally, age-of-(onset) acquisition of the non-societal language (AoA) and length of exposure to the non-societal language (LoE) were also measured. We observed a mean score of 10.46 for Social (L2 learners = 9.84, SD = 8.69; HSs = 11.16; SD = 9.13), a mean score of 1.59 for Home (L2 learners = −5.66, SD = 3.01; HSs = 9.86; SD = 5.77), and a mean score of 0.71 (L2 learners = 0.70, SD = 1.88; HSs = 0.72; SD = 1.51) for Proficiency. Participants also completed the LexTALE (Lemhöfer and Broersma, 2012) to assess general English proficiency (L2 learners = 68.75%, SD = 12.40; HSs = 65.27%, SD = 12.46) (see Supplementary Table 1 for all participants' metadata), for which the groups did not differ [*t*_(57)_ = 1.08, *p* = 0.28].

### Study procedure

The research procedures in this study were approved by the University of Konstanz Research Ethics Committee. Before taking part in the experiment, participants gave written informed consent and confirmed no contraindication to the EEG investigation. Participants who presented a neurological condition (e.g. epilepsy, multiple sclerosis, etc.) where excluded from this study. Furthermore, participants were compensated for their time. First, participants completed the LSBQ. Then, for the EEG recording session, participants were fitted with an appropriate actiCap in accordance with the 10–20 system (Brain Products, Inc). The experiment was presented on a 17-inch screen. The Flanker task (Eriksen and Eriksen, 1974) was administered using Presentation software (Presentation^®^, Neurobehavioral Systems). Participants were presented with sets of five arrows and asked to specify the direction of the arrow in the middle. Surrounding arrows (flankers) were pointing either in the same direction (congruent condition: <<<<<) or in a different direction (incongruent condition: <<><<). Incongruent trials require participants to ignore the conflicting information coming from the surrounding arrows. Thus, comparatively, incongruent conditions involve greater recruitment of the executive control system.

The task procedure was first explained to the participants. A brief practice session preceded the experimental blocks to allow the participants to familiarize themselves with the task. The practice session consisted of a total of 12 trials, 4 congruent trials, 4 incongruent trials and 4 neutral arrows (only a single arrow was shown, pointing either left or right, this condition was not used in the EEG analysis). Participants were instructed to use their corresponding index finger to press left (on a button box—RB-740, Cedrus^®^) if the central arrow (or the single arrow in the neutral trials) pointed to the left, and to press right if the opposite was true. Participants were instructed to be as quick and accurate as possible. The experimental session consisted of two blocks of 120 trials each presented in a randomized order: 40 incongruent trials, 40 congruent trials and 40 neutral trials. Thus, the total number of trials was 240 (80 trials per condition). Each trial began with a fixation cross in the middle of the screen presented for a jittered duration of 400–1,600 ms (at randomized steps of 100 ms). The fixation cross allowed the participants both to focus their attention on the center of the screen and to reduce saccadic eye movements. Afterwards, a 200 ms baseline blank screen appeared followed by the stimulus, which was presented until the participants responded or for a maximum duration of 1,500 ms. An inter-trial interval (ITI) blank screen of 2,000 ms followed to avoid eventual carryover effects. The participants were instructed to take a short break between trial blocks. The EEG signal was continuously recorded from 32 Ag/AgCl scalp electrodes (LiveAmp32, Brain Products, Inc). AFz acted as the ground electrode and FCz as the online reference. The Fp1 and Fp2 electrodes, located on the forehead above the eyebrows, were employed to detect and monitor vertical and horizontal eye movements. Impedances were kept below 25 kΩs. Data were recorded with an online filter of 0.01–200 Hz and was amplified and continuously digitized at a 1000 Hz sampling rate using a Brain Vision LiveAmp amplifier.

### Data pre-processing and time-frequency analysis

Offline processing of the data was done in two steps. First, in Brain Vision Analyzer (BVA) 2.0 (Brain Products, Inc), data were band-pass filtered from 0.1–45 Hz^1^. Then, the signal was segmented from −750 to 1,250 ms around the stimulus onset. Next, an automatic independent component analysis (ICA) implemented in BVA was used to detect and eliminate eye movements and blinks. ICA was performed on the segmented dataset with 512 steps and an infomax (Gradient) restricted algorithm. A spheric spline topographic interpolation was employed if anything unusual (e.g., high noise, electrode picking up the heartbeat signal, etc.) happened to the electrodes during the recording (1 to maximal 3 electrodes per participant - total number of interpolated electrodes = 0.01% of the dataset). All trials were manually inspected for artifacts (drifts, excessive muscle artifact, blocking, etc.). Trial rejection resulted in the exclusion of 226 trials (0.016% of the data). The remaining epochs were baseline-corrected (-100 ms prior to stimulus onset) and then re-referenced to the averaged mastoids (TP9/10). Data were then exported for time frequency analysis using the Fieldtrip toolbox implemented in Matlab (Oostenveld et al., 2011). Only items correct at the behavioral level were used for further preprocessing and analysis.

The power spectrum was computed in the 2–45 Hz frequency range to accommodate the following frequency bands: 4–7 Hz (theta frequency), 8–12 Hz (alpha frequency), 13–20 Hz (low beta frequency), 21–30 Hz (high beta frequency) and 31–45 Hz (gamma frequency). To calculate the power spectrum, a 500 ms long stable moving window and a Hanning taper were employed. Power changes were computed in steps of 50 ms and 1 Hz. Given the properties of the time-frequency analysis and the BVA to Fieldtrip export procedure, the resulting time-frequency representations (TFRs) contained data points between 500 ms prior to and 950 ms after the stimulus onset. These TFRs were expressed as a relative change from the −500 to −100 ms baseline period. Finally, TFRs were averaged for each subject and separately for each of the three conditions. For the remainder of the analysis, both at the brain and behavioral level, the interference effect was used, i.e., the difference between incongruent and congruent trials.

### Statistical data analysis

#### Behavioral analysis

For the analysis of reaction times (RTs), RTs lower than 200 ms and non-accurate were excluded from further analysis. This led to the removal of 139 trials (0.97% of the trials). Repeated measures ANOVAs were performed for RTs (factors: Group [heritage speakers (HSs), L2 learners (L2)] × Condition [incongruent, congruent, neutral]. For the accuracy analysis (Acc), generalized linear models from the binomial family were employed looking at the fit of condition and group on accuracy [glm(accuracy ~ condition^*^group, family = binomial].

#### Time-frequency representation analysis

Analyses were performed on total oscillatory activity. Two sets of statistical analyses on the EEG data were performed in Fieldtrip: the first was aimed at comparing the interference effect between the two language groups (testing the first hypothesis, i.e., between group differences), the second was aimed at identifying the time-frequency clusters of interest for further analysis of individual differences (testing the second hypothesis, i.e., related to individual differences). A 1,000-randomizations cluster-based permutation approach (see Maris and Oostenveld, 2007) was used (two tailed dependent t-tests, cluster alpha = 0.05). The tests were run on the entire post-stimulus onset window with averaging per frequency band. A statistical threshold of *p* < 0.025 per tail was used to compute *t*-values for every electrode-time-frequency point and for corrected cluster-level significance.

##### Between-group comparison of the interference effect

To compare the interference effect between the two groups, we first subtracted the power spectrum for the congruent condition from the incongruent condition separately for the HS and L2 groups. We then ran a cluster-based permutation test comparing the resulting power spectra between them.

##### Identifying time-frequency clusters of interest

**Figure 1:**
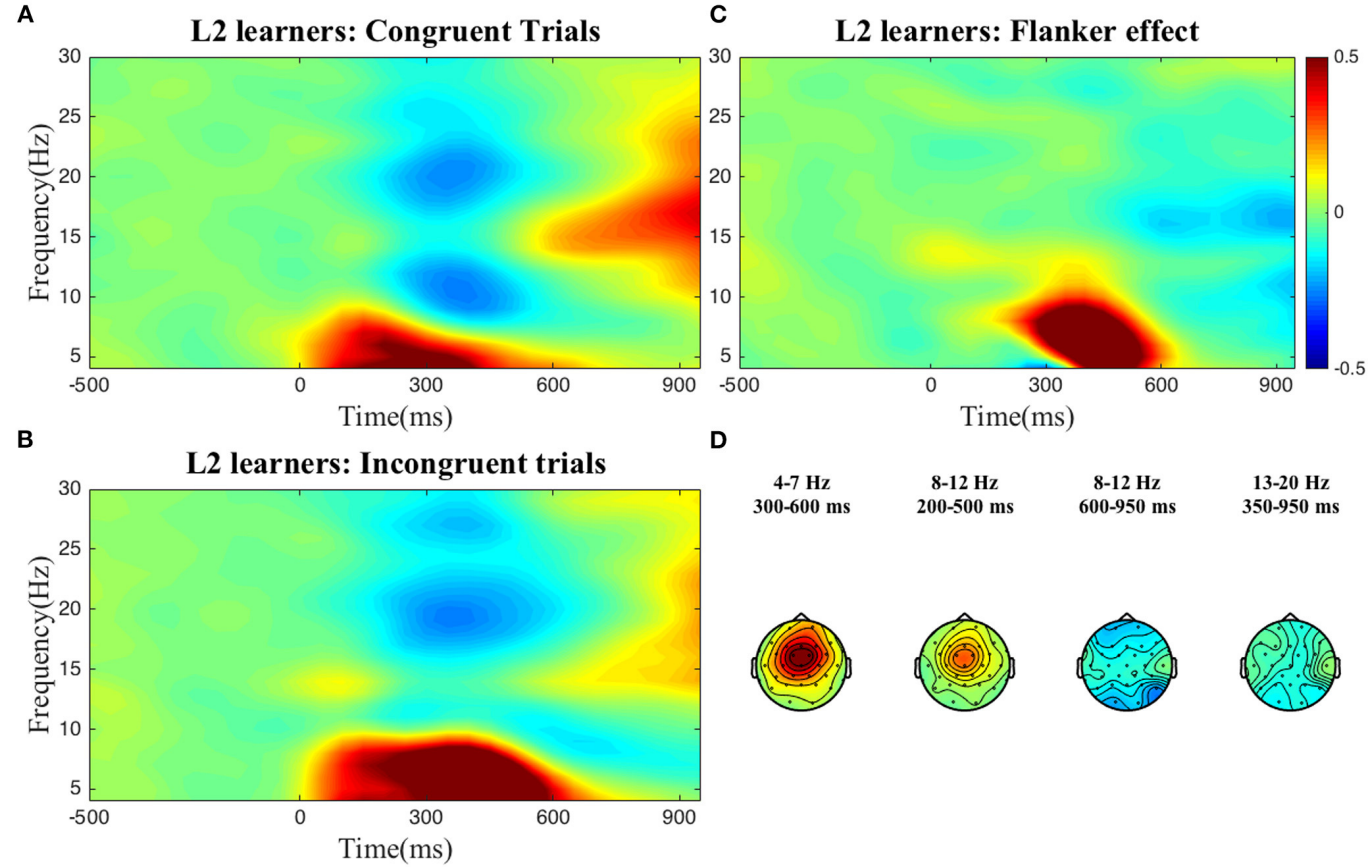
TFRs for the L2 learner group. Averaged time-frequency representations of individual conditions **(A,B)** and the Flanker effect **(C)** for the L2 learners for 4–30 Hz in a representative electrode, Cz and topographical plots **(D)** of the Flanker effect for the time-frequency clusters of interest. The Flanker effect was computed by subtracting power in the Congruent condition from power in the Incongruent condition. The color bar applies to both single electrode and topographical plots and indicates relative power change.

**Figure 2:**
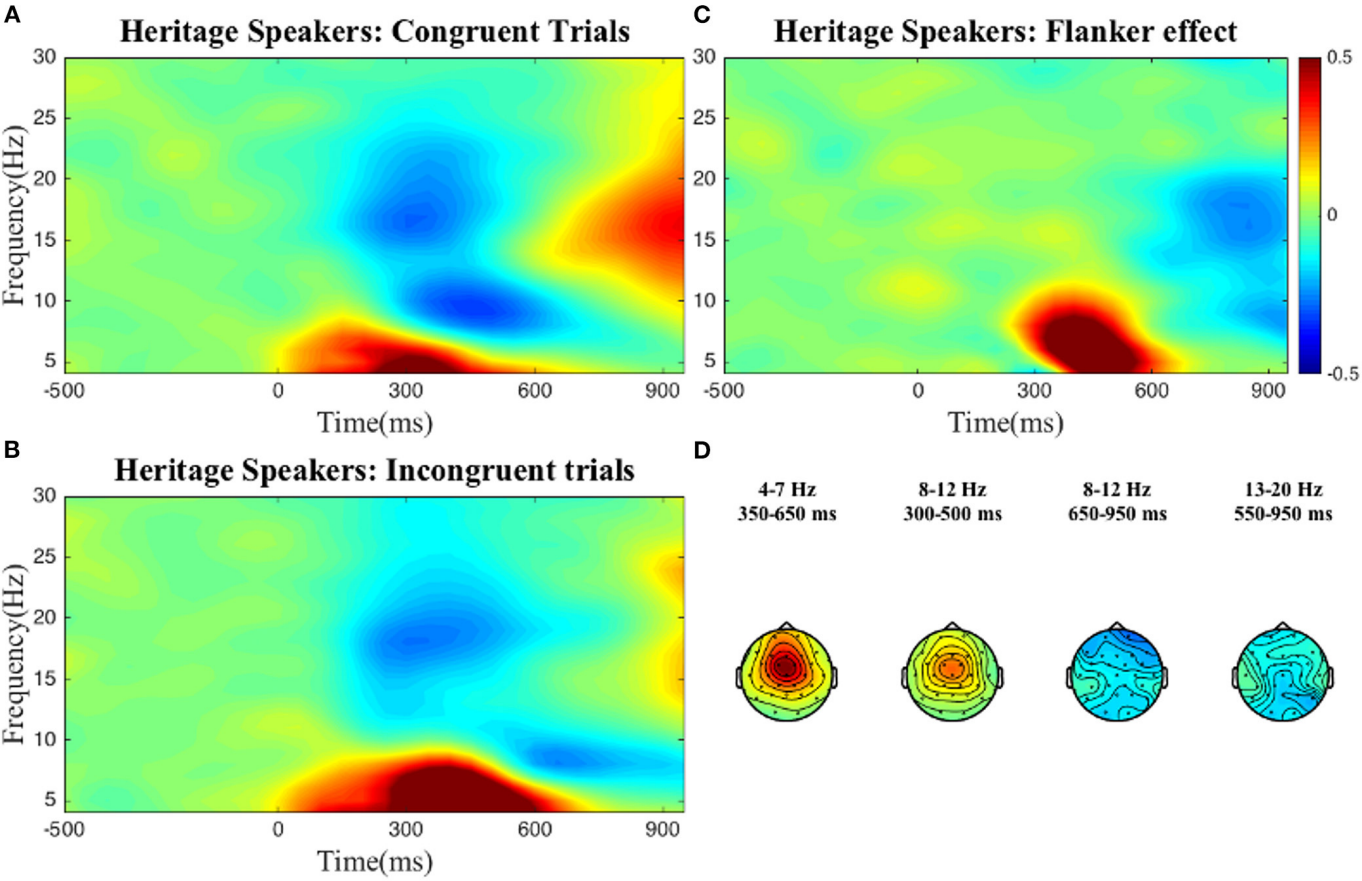
TFRs for the HS group. Averaged time-frequency representations of individual conditions **(A,B)** and the Flanker effect **(C)** for the heritage speakers for 4–30 Hz in a representative electrode, Cz and topographical plots **(D)** of the Flanker effect for the time-frequency clusters of interest. The Flanker effect was computed by subtracting power in the Congruent condition from power in the Incongruent condition. The color bar applies to both single electrode and topographical plots and indicates relative power change.

To select the time-frequency-electrode clusters of interest (scalp electrode region) for further analysis of individual differences, we compared power between the incongruent and congruent conditions (interference effect) in the frequency bands described above. For reasons discussed below, this process was done at the group level, i.e., for both groups separately, and at the sample level, i.e., collapsing all participants into one group. As can be seen in the figures below, at the group level, statistically significant condition differences were found within the following clusters: a positive theta cluster (300–600 ms) across fronto-central electrodes, a negative alpha cluster (600–950 ms) and positive alpha cluster (200–500 ms) over broad frontal electrodes, and a negative low beta cluster (350–950 ms) across posterior electrodes for the *L2 learners* (Figure 1). For the *HSs* (Figure 2) the following pattern emerged: a positive theta cluster (350–650 ms) across fronto-central electrodes, a broadly distributed negative alpha cluster (650–950 ms), a positive alpha cluster (300–500 ms) in fronto-central electrodes, and a broadly distributed negative low beta cluster (550–950 ms). At the *whole collapsed sample level* (both bilingual type groups included), the following clusters were found: a positive theta cluster (300–650 ms) across fronto-central electrodes, a broadly distributed negative cluster (600–950 ms) and a positive (350–500 ms) alpha cluster across fronto-central electrodes, a broadly distributed negative low beta cluster (450–950 ms) and a negative high beta cluster (750–950 ms) over central-posterior electrodes. These clusters were extracted and used for the computation of the average power-individual-frequency band.

#### Individual differences, language experience factors and brain interaction

A multiple regression analysis was conducted, extracting individual power values (for each frequency band of interest) correlated to the language variables derived from the LSBQ: non-societal language exposure and use at home (NSL-Home), non-societal language use in the society or community (NSL-Social), proficiency in the societal language (Proficiency), Age of L2 or 2L1 onset (AoA), and Length of exposure to the non-societal language (LoE). Biological age (Age) at time of testing and LoE highly correlated (*r* = 0.95, *p* < 0.001). For this reason, we decided to drop LoE from our models. We also included Sex (male, female) and socio-economic status (SES) as covariates. All continuous variables included in this and following models were centered around the mean. Treatment coding was applied to categorical variables. The process was done in two parts. First, we correlated the language variables to the complete brain collapsed sample (linear regression models) (see Section **Identifying time-frequency clusters of interest** for clusters of interest). Then, we looked at individual correlations between these language experience factors and neural oscillations within each group separately (linear regression models) (see Section **Identifying time-frequency clusters of interest** for clusters of interest). All models were performed using the *lmrob* function from the *robustbase* package (Yohai, 1987; Koller and Stahel, 2011) on the statistical software R (R Core Team, 2021). Robust functions are designed to be more sensitive to outliers and suboptimal normal residuals, which is often the case with brain data.

#### Interaction brain and behavioral analysis

For this analysis, the interrelationship between brain oscillations and behavioral interference was investigated, i.e., how neural correlates predict response speed differences between incongruent and congruent trials (testing the third hypothesis). To do so, we used the data at the *whole collapsed sample level* (both bilingual type groups included) (see Section **Identifying time-frequency clusters of interest** for clusters of interest).

## Results

### Behavioral results

A summary of the behavioral results values (mean accuracy and reaction times) is shown below (Table 1). The accuracy analysis revealed that generally both groups performed better in the congruent conditions in comparison to the incongruent condition (*E* = 1.91, *SE* = 0.44, *p* = 0.0002 for the L2 learners and *E* = 2.80, *SE* = 0.52, *p* = <0.0001 for the HSs) and neutral (*E* = 1.54, *SE* = 0.28, *p* = <0.0001 for the L2 learners and *E* = −1.99, *SE* = 0.36, *p* = <0.0001 for the HSs). Differences between the groups were only found in the incongruent condition (*E* = 0.62, *SE* = 0.20, *p* = 0.03), where the L2 learners were more accurate. In the analysis on RTs, there was a main effect of condition [*F*_(2,174)_ = 119.172, *p* = <0.0001]. Both groups were faster in the congruent and neutral conditions in comparison to the incongruent condition (see Table 1), but there were no group differences in any of the conditions. Furthermore, no language group by condition interactions where found.

**Table 1:** Summary of the behavioral analysis (mean Reaction Times and Accuracy) for the L2 learners (*n* = 32) and HSs (*n* = 28) for the three different conditions (congruent, incongruent, and neutral) in the Flanker task.

**Condition**	**Group**	**meanRT (ms)**	**sdRT (ms)**	**meanAcc**	**sdAcc**
Congruent	L2 learners	450	81.6	0.998	0.048
	HSs	436	89.1	0.998	0.042
Incongruent	L2 learners	560	123	0.984	0.124
	HSs	561	126	0.971	0.167
Neutral	L2 learners	434	80.2	0.994	0.079
	HSs	418	80.8	0.996	0.063

### Time-frequency results; between-group comparison of the interference effect

We found no differences between the two groups, i.e., at the aggregate level HSs and L2 learners displayed very similar brain oscillatory patterns when looking at interference suppression in the Flanker task (see Figures 1, 2).

### Individual differences, language experience factors and brain interaction

Although there do not seem to be any aggregate differences, this does not mean that individual level factors are not at play, whether in a collapsed group of all participants or across participants in each group separately. In principle, it is possible that each group contains participant samples with similar degrees of relevant experiential variation washing out any obvious effect. We cannot discount *a priori* that individual linguistic experience/engagement could trump any potential effect AoA and/or -lingualism status (bi- vs. multilingualism) might confer independently. In other words, it might be the case that timing (onset and duration) of multiple language experience and/or quantity of languages (bilingual vs. multilingual) offer no explanatory value above and beyond individual engagement with multiple language use whenever one attains competence in more than one language. If so, this would mean that there are no caveats to the claim that linguistic engagement patterns are primarily deterministic for neurocognitive outcomes. Afterall, it is not only conceivable but indeed realistic to find adult L2 learners who are much more engaged in bilingual experiences than some childhood simultaneous bilinguals, even if the general trend and intuition pushes us to think in the opposite direction. If on the right track, there would be no reason to treat HSs and L2 learners distinctly. Of course, this is an empirical question. One, we submit, of significant importance that our design allows us to address. To test this, we first collapsed the two groups and ran multiple regressors to investigate how language usage variables predict neural outcomes overall. Some interesting results emerged significant, yet only for low beta. However, the question remained as to whether or not collapsing is the best approach. If it turns out to be that group categorizing variables (e.g., AoA) brings something to bear independently, probing for individual differences related to linguistic experiences would still make sense to do regardless. However, doing so within each group separately should prove more meaningful.

**Figure 3:**
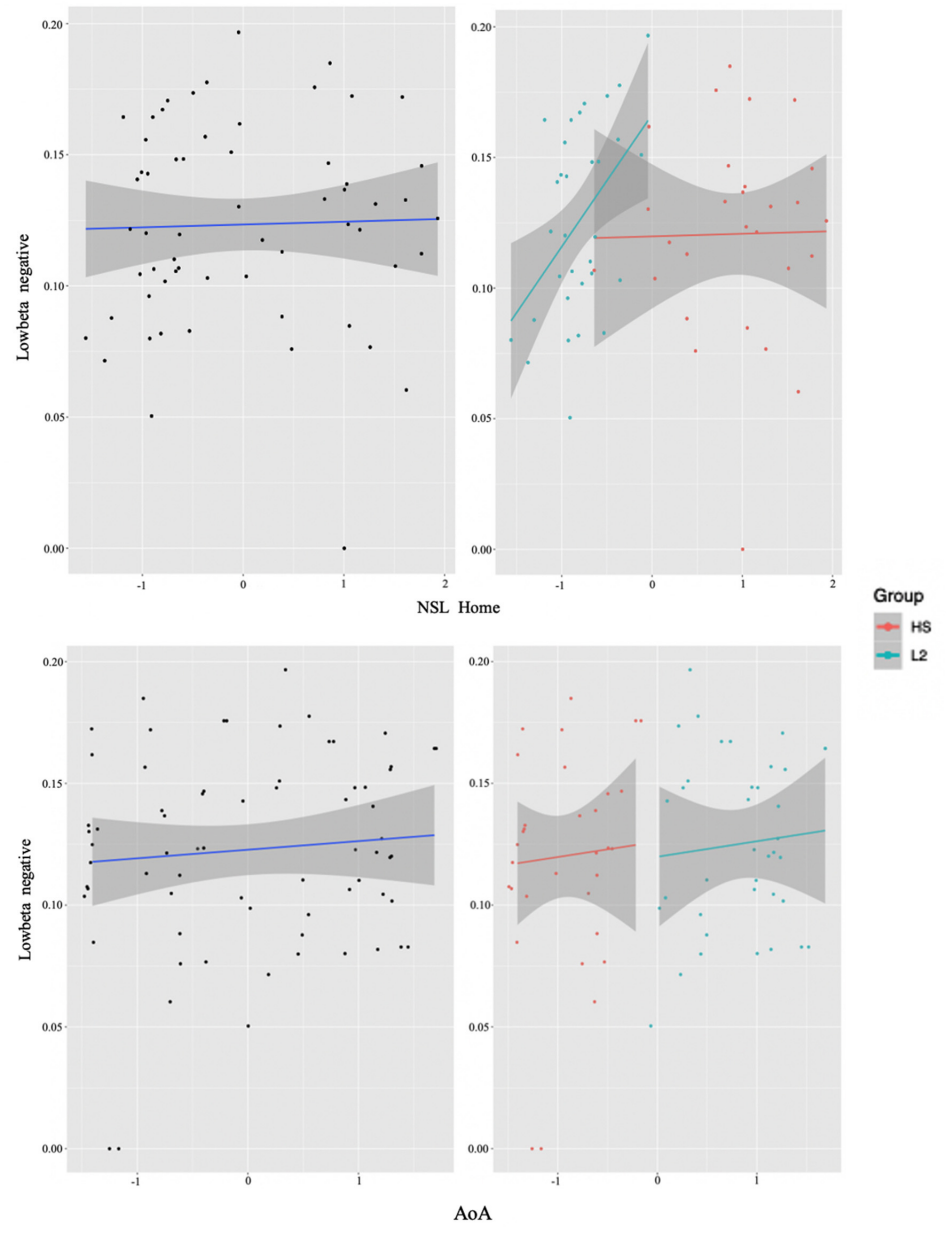
Collapsed group analysis results. The two figures on the left represent the correlation between experience factors (NSL Home top and AoA bottom) and low beta power from the whole collapsed dataset (units on both axes are normalized ones: *x*-axes age and *y*-axes power) (see Section **Identifying time-frequency clusters of interest** for clusters of interest). In the two right figures, the individual data points, representing the participants, have been sorted out into the two groups (HS for the heritage speakers of Italian and L2 for the L2 learners), where again the NSL Home interaction is on the top and AoA on the bottom. Notice how the two groups cluster separately, with little to no mixing between them. In the two right figures, the L2 learner group (L2) is represented in blue and the HS group (HS) in red.

Upon careful inspection of the data, it became clear that when collapsed together the two groups were clustering separately, instead of forming a homogenous bilingualism continuum (see Figure 3 for an example of this phenomenon). Therefore, the preferred analysis was the one that treated the groups separately. Moreover, upon treating the groups separately we note that significant effects are more pervasive, that is, not relegated only to low beta.

**Figure 4:**
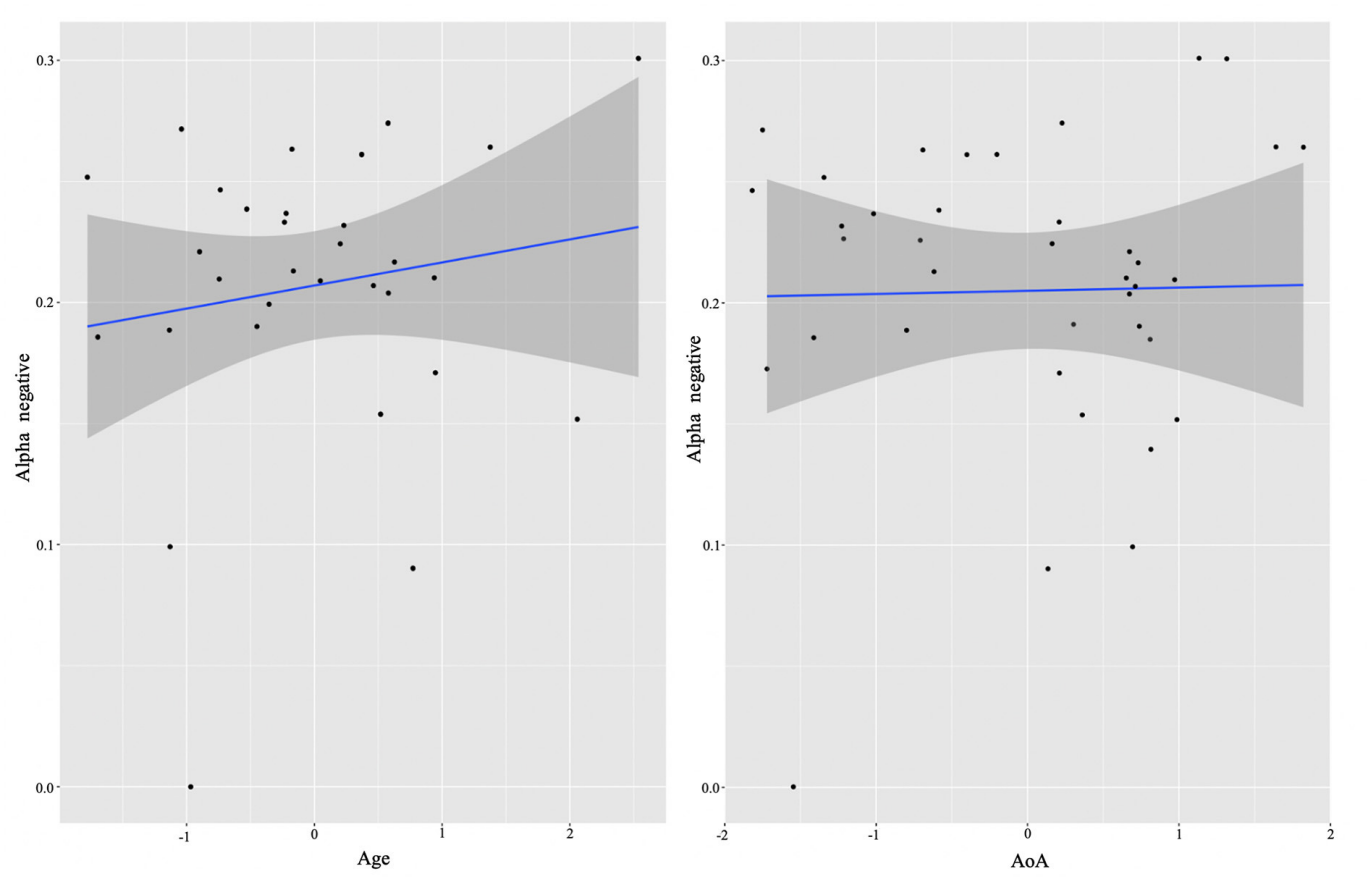
L2 learner group individual analysis results in alpha. Predicted values of Age (left) and AoA onset (right) on alpha power (negative cluster) in the L2 learner group (see Section **Identifying time-frequency clusters of interest** for clusters of interest).

**Figure 5:**
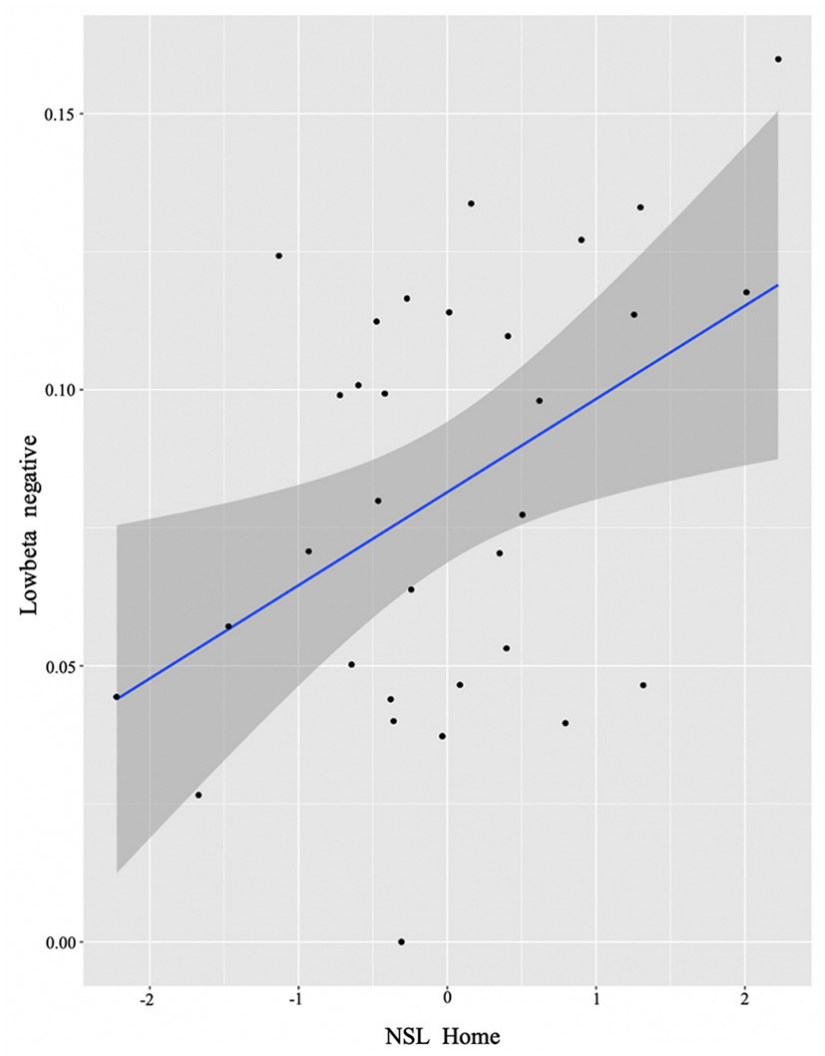
L2 learner group individual analysis results in low beta. Predicted values of NSL Home on low beta power (negative cluster) in the L2 learner group (see Section **Identifying time-frequency clusters of interest** for clusters of interest).

In order to examine whether language experience modulates the magnitude of power for each group, we again ran multiple regression analyses for each cluster. We report only significant results. Starting from the L2 learners, we found effects in alpha (negative cluster) and low beta (negative cluster) frequencies, and exclusively positive correlations with the experience factors. The significant background-related factors that predicted power in the alpha cluster were AoA (*E* = −0.02, *t* = −2.17, *p* = 0.04) and Age (*E* = 0.03, *t* = 2.33, *p* = 0.03) (see Figure 4), whereas NSL-home (*E* = 0.02, *t* = 2.47, *p* = 0.02) significantly correlated with low beta (Figure 5).

**Figure 6:**
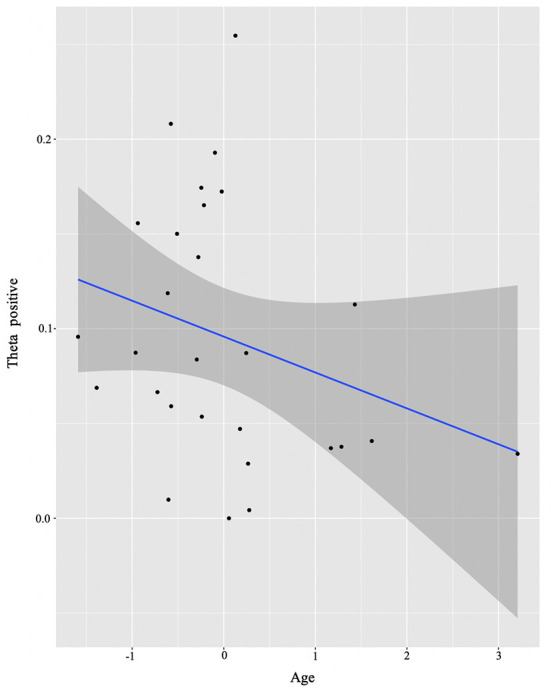
HS group individual analysis results. Predicted values of Age on theta power (positive cluster) in the HS group (see Section **Identifying time-frequency clusters of interest** for clusters of interest).

For the HS group, we only found a negative correlation (Figure 6) between Age and theta power (positive cluster) (*E* = −0.03, *t* = −2.52, *p* = 0.02).

### Brain and behavior interaction

**Figure 7:**
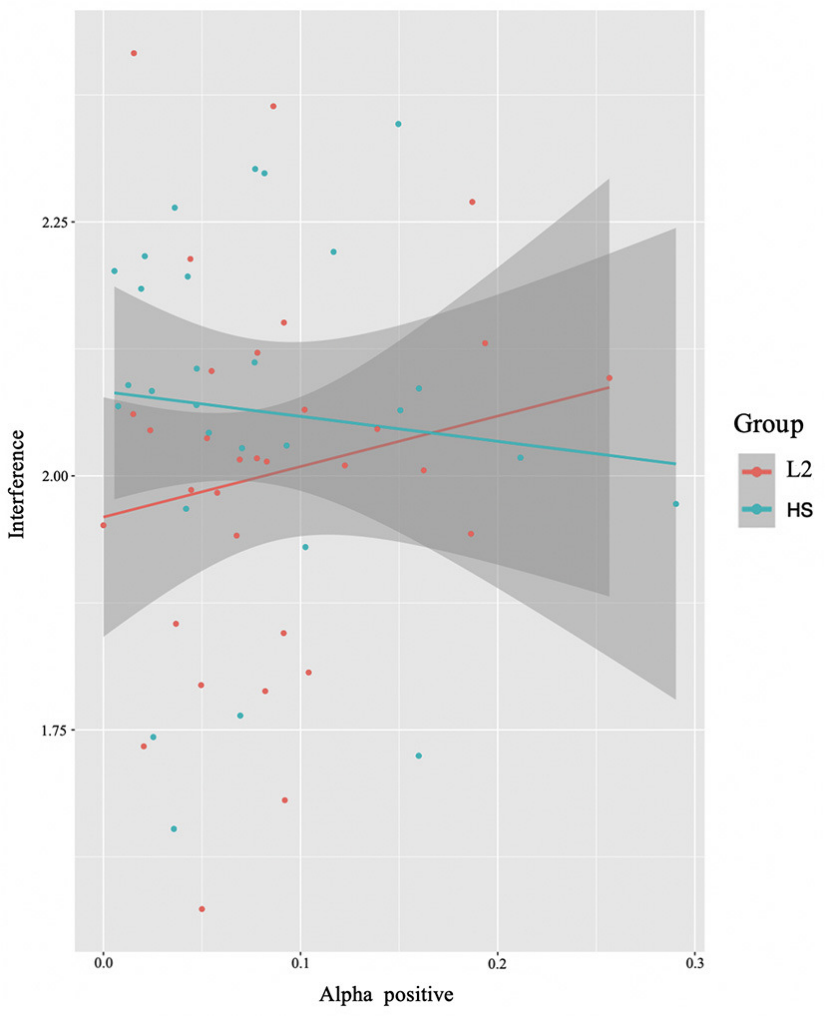
Brain x behavioral analysis results. Interaction between alpha power (positive cluster) and group and interaction with behavioral RTs interference. The L2 learner group (L2) is represented in red and the HS group (HS) in green (see Section **Identifying time-frequency clusters of interest** for clusters of interest).

In this analysis, we modeled RTs interference as predicted by brain-data^*^group. We found a significant interaction between group and alpha power (in the positive cluster) (*E* = −0.99, *t* = −2.06, *p* = 0.04) (Figure 7). Interestingly, the two groups show opposite patterns. While a positive correlation is found between alpha power and interference suppression effect for the L2 learners, the HSs exhibit a negative correlation: the more alpha was recruited, the smaller the difference was between incongruent and congruent trials.

## Discussion

In what follows, we unpack and interpret the above presented results in general and specifically in relation to our three hypotheses outlined in the introduction.

### Between-group comparison of the interference effect

The first hypothesis anticipated the possibility that the L2 learner group would exhibit increased theta activation whereas, conversely, the HS group might show increased alpha suppression for inhibitory control as a result of the inherent difference in duration (and context) commensurable with bilingual type (DeLuca et al., 2020). The logic was as follows: adaptations to control demands (such as those associated with bilingual experience) are predicted to initially manifest in fronto-cortical regions and networks (Grundy et al., 2017; Pliatsikas, 2020), linked to increased theta band activation in tasks measuring processes related to inhibitory control (Cavanagh and Frank, 2014; Duprez et al., 2018; Brunetti et al., 2019; Pscherer et al., 2021). Given this, we might have expected that the shorter duration of bilingual experience associated with the L2 learner group to manifest as greater reliance on fronto-cortical circuits to handle inhibitory control demands, resulting in greater theta band activation. As discussed in the introduction, here we are referring to absolute (quantitative) as well as relative (qualitative) “time”, given that the L2 English is not within immersion, but the HS experience entails intensive early immersion in two languages by definition^2^. Alternatively, the longer timeframe (and intensity) of exposure to (at least) two languages inherent to the HS context would show a shift in reliance toward subcortical circuits to handle these control demands more efficiently (Grundy et al., 2017; Pliatsikas, 2020), which would result in stronger modulations in alpha power suppression (Mazaheri et al., 2014).

However, our findings show no such differences, disconfirming our first prediction. This, of course, does not mean that all bi-/multilinguals are the same. It is simply the case that our HSs, whose timing and intensity to bilingualism in early childhood tangibly differed, were not distinct in the aggregate from the L2 learners studied herein. Whereas, frameworks like UBET (DeLuca et al., 2020) and the *Bilingual Anterior to Posterior and Subcortical Shift* (BAPSS) (Grundy et al., 2017), from which we derived the predictions, anticipate such a distinction, neither make reference to the timeframe under which adaptations toward efficiency occur. Keeping in mind, then, that our L2 learners are not only proficient in English but have had significant time with English competence it could simply be that they are past a stage in bilingual development where increased reliance on fronto-cortical circuits is implicated. In other words, for all intents and purposes any potential effect duration of bilingualism could have had is surpassed in these groups. Without a monolingual comparison or an additional bilingual group with less L2 experience (beginners) we cannot tease the following three possibilities apart: (i) bilingualism has no such effect here (unlikely in light of the rest of the data unpacked below), (ii) there simply is no difference between L2 learners and HSs *par excellence*, that is, at any stage of development or (iii) such differences would obtain, but only when L2 learners are under a particular threshold of bilingual experience. Ultimately, it might not have been a fair question to ponder from the outset since HSs tested in adulthood are inherently at a mature state of linguistic bilingual development, whereas L2 learners at similar ages are at various stages of linguistic development—i.e., with HSs we are looking at neurocognitive effects of bilingual language maintenance as opposed to bilingual language development as one can do at various stages of proficiency with L2 learners.

Beyond the aspect of AoA, which admittedly is confounded in our HSs with number of languages as a consequence of the reality of HS bilingualism in the European context, one might ask if multilingualism over bilingualism evidences any differences as well. Recall that our design and specific sampling matched the HSs and L2 learners in English and German proficiency as well as dominance (they are all German dominant). Thus, linguistically what differentiated them was the presence or absence of an additional, heritage Italian, grammar in the mind. Given our results, one might conclude multilingualism over bilingualism and, more specifically, heritage language experience *per se* is not deterministic. Any such definitive conclusion at this point, however, would be precipitous based on this initial analysis alone. As we will unpack below, such a conclusion is, in fact, challenged by other aspects of the present data. Given the nature of our data—the mere reality of HS bilingualism in Europe where English is not the societal language but ubiquitous—we will not be able to tease apart AoA from multilingualism with our HSs. Notwithstanding, we suspect that AoA rather than multilingualism *per se* or an interaction of the two is the driving force of the differences noted in our results that we elaborate on below, not least in the context of other studies showing AoA effects on neurocognitive adaptations likened to bilingualism (e.g., Luk et al., 2011; Tao et al., 2011; Delcenserie and Genesee, 2017, see Berken et al. (2017) for a review of MRI-based AoA supportive evidence).

In truth, little is known about the processes of neurocognitive adaptations to multilingualism as a *departure* from bilingualism; that is, if and how the brain further adapts to the acquisition and management of a third (or more) language. It is intuitive, if not theoretically reasonable, to anticipate that adding more languages to the brain/mind systems would increase control and processing demands for successful language selection and use. After all, there is no shortage of literature showing that multilingual acquisition and processing are distinct from bilingualism in linguistic domains (see for review Rothman et al., 2019). However, it is equally reasonable to expect that three or more languages would bring nothing or very minimal effects to bear on domain general neurocognitive adaptations specifically above and beyond two. This could be so either because the change in state in the relevant sense from bilingualism to multilingualism is much less severe than it is from monolingualism to (at least) bilingualism or because there are ceiling effects for relevant adaptations that are already met by managing two languages, at least under certain conditions. In other words, the brain might show highly differential adaptations when moving from a one-language system to a two-language system, but these adaptations may not be as required (or visible) when moving to a multi-language system. In this light, individual differences in language experiences, rather than number of languages spoken, are likely to drive patterns of multilingual neurocognitive adaptation (see Yee et al., 2022), to which we now turn.

### Individual differences in language experiences

To test our second hypothesis regarding individual difference correlations to bilingual experience, we ran a cluster-based permutation analysis to explore the neural mechanisms accompanying conflict resolution, extracting power values per condition and regressing them with continuous measures of bilingual experience. For reasons discussed above, we first did this in a combined sample of the two groups, then by analyzing them separately. Results suggested that AoA of the L2/2L1 and usage of the non-societal language at home positively predicted low beta recruitment for the whole group (see Figure 3). However, when we plotted this while annotating individuals by group, it became visually apparent that only one of the two groups (the L2 learners) was driving the non-societal language effect and, overall, that the groups were not mixing into the continuum of individual experiences. Therefore, we will not discuss the results in terms of the combined group. Alternatively, we re-ran the analysis separating out the two original groups not least because previous work clearly indicates that both bilingual types, on their own, could potentially display within-group individual variation (Kupisch and Rothman, 2018; DeLuca et al., 2019a, 2020).

To the extent that this approach panned out, as in our analysis below, there is a combined epistemological and empirical note of importance. It can be true that bilingual experience is deterministic for outcomes of neurocognitive adaptations in general but teasing this out can be inadvertently obscured by combining proverbial apples and oranges. Combining distinct types of bilinguals, as in the whole group analysis, with crucial variables that make them incomparable at some level of apparent importance, such as AoA, could interact with how experience is differentially uptaken in each bilingual type and thus introduce noise. In other words, experience can matter generally, but not necessarily play out in the same ways depending on how experience interacts with other features. If so, we might expect to see that variation in experience predicts outcome differences regardless of bilingual type, but is appreciated only in comparison to peers within a singular type (at least for some outcomes whereas others might not show a type division). Conversely, it could be the case that experience only matters when certain other variables are true, for example, only if bilingualism commenced before a particular age. Maintaining group distinctions then, as warranted by our first pass analysis, will ultimately help us to determine this. Perhaps then, the order in which we approached these analyses should have been reversed. Examining the groups separately and showing the same trends in each would have certainly warranted a follow up to see if the same held true when combining both groups: a lesson learned should also be a lesson shared.

Returning to the two-group regression analysis, let us remind the reader which clusters emerged for both the HS and L2 learner groups: (i) a positive theta cluster, (ii) an early positive alpha cluster, (iii) a late negative alpha cluster and (iv) a negative low beta cluster. These clusters are in line with previous literature on the oscillatory dynamics of domain general cognitive control. Increased theta power in the incongruent relative to the congruent conditions reflects greater conflict resolution demands (e.g., Nigbur et al., 2011). Both alpha decreases and increases in incongruent relative to congruent trials have been reported previously (McDermott et al., 2017). These increases and decreases have different neural generators. For example, McDermott et al. (2017) localized alpha decreases to the parietal and occipital cortices as well as the areas near the right temporoparietal junction (TPJ) with these latter areas being a part of a ventral attentional network believed to facilitate the detection of the stimulus, especially in the presence of unexpected or distracting stimuli. Alpha power increases were found in the part of the dorsal attention network which supports the selection of sensory stimuli and their linking to the appropriate motor response (McDermott et al., 2017). Given the role of beta power for motor response preparation and execution (Engel and Fries, 2010; Grent-'t-Jong et al., 2013), the relative decrease of beta power for the incongruent relative to the congruent conditions could reflect conflict at the response level, that is simultaneous activation of both response directions in incongruent trials compared to the single direction in congruent trials.

Thus, qualitatively the HSs and L2 learners in their aggregates exhibited similar neural signatures of inhibitory control. However, in line with hypothesis 2, further analysis of individual differences revealed that these effects were differentially modulated by experiential factors for the two groups. For the L2 learners, a negative alpha cluster positively correlated with chronological Age (which, given the relatively small difference in age between the participants, can be considered as a proxy for the duration of exposure) and AoA. That is, the older the participants were at time of testing (hence the more exposure they had to the L2) and the older they were when they acquired their L2, the smaller the alpha power decrease accompanying conflict resolution was. Under the interpretation that reduced alpha suppression reflects more efficient task performance (Zhuang et al., 1997; Riečanský and Katina, 2010; Kielar et al., 2014), the Age effect suggests that participants who were exposed to their L2 longer demonstrated less effortful processing of incongruency in the Flanker task. This result is in agreement with the theoretical suggestion that longer duration of dual language use (which implies prolonged juggling of two language systems) confers adaptations that afford more efficient and automated processing of conflicting information (Grundy et al., 2017; Pliatsikas, 2020). However, as discussed in the introduction, there is also an account predicting that prolonged duration of bilingual experience would be associated with increased alpha suppression in posterior and subcortical areas in situations of control demands (DeLuca et al., 2020). This prediction is more in line with our finding of positive correlation between the AoA (given that it is often confounded with prolonged duration of bilingual use) and alpha decrease, whereby the earlier the participants learned their L2, the stronger alpha decreases are exhibited. Thus, the effects of Age and AoA are somewhat contradictory in our study and further work should more closely examine the contributions of these different but related factors.

Additionally, within the L2 group, the negative low-beta cluster positively correlated with the use of non-societal language at home, namely the more English is used at home, the smaller the relative beta power decrease is. Although at first glance this result might seem controversial, when considering the use of English at home in a society like Germany in younger populations, we can easily pinpoint factors of language-use divergence across individuals (some having international roommates or friends, playing online games, differential usage of social media and others). And so, similarly to the alpha power results, such reduced beta power may indicate more efficient handling of conflicting information (Kielar et al., 2014) by individuals who more intensely use their L2. This is in line with the account suggesting that a change in control demands is expected with increased intensity of L2 use (DeLuca et al., 2020).

For the HSs, Age at time of testing negatively correlated with power in the theta frequency band, i.e., the older the participants were (hence, the more exposure to Italian and English, and thus prolonged exposure to multiple languages), the less theta power they recruited for processing incongruency. The reduction of theta power as a function of duration of exposure might signal a reverting to baseline levels of the functional recruitment patterns in fronto-cortical regions resulting from the increased efficiency in juggling their languages and a reduction in inhibitory control demands (DeLuca et al., 2020; Pliatsikas et al., 2021). So how can it be that given very similar brain signatures of conflict resolution between the two groups, we find these signatures to be differentially modulated by language experience factors? Here is where we believe we see an effect of HS bilingualism itself. Recall that the groups are controlled for context of learning and proficiency of English. However, the HSs experience with Italian under an immersion context, i.e., their earlier intensity to naturalistic bilingualism, drives differential outcomes.

The groups did not overlap on the continuous measures of AoA and the use of non-societal language at home. Thus, it could be that while these distinctions in experience do not lead to qualitative differences in which neural operations are recruited for processing conflict (at least the kind of conflict induced by the Flanker task), they do define the ways in which they interact with experiential factors. It could also be the case that when more than one language is acquired early enough and/or when the non-societal language use at home is more intense, there are threshold effects related to experiential factors (e.g., duration of exposure) differentially triggering conflict detection and the communication of the subsequent need for enhanced control (Cavanagh and Frank, 2014). When the onset of exposure to another language occurs past a certain threshold (e.g., as in our L2 learners) and non-societal language is minimally or not used at home, the duration of exposure may not have that same effect. However, in the case of later onset of exposure to L2 and minimal use of non-societal language at home, experiential factors modulate the engagement of attentional mechanisms required for managing the conflict as reflected in alpha oscillations and recruitment of the motor system reflected in beta oscillations.

The current study does not allow us to disentangle the effects of AoA and non-societal language use at home on the relationship between experiential factors and neural signatures of inhibitory control. It is possible that the differences observed between the two groups are driven by one or both of these measures, or that adaptations required by each are likely to be modulated by the other (DeLuca et al., 2020). Further work should address this issue by carefully selecting appropriate test populations to address this query, for example, by adding a group of multilinguals with the same languages to our HS group but have acquired their L2 and L3 in adulthood (e.g., German natives who acquire English in the typical (early L2) case and are L3 learners of Italian with high proficiency).

### Brain and behavioral interaction

As per hypothesis 3, we investigated the anticipatedly observable relationship between oscillatory dynamics and reaction time (RT) performance, inclusive of whether the pattern was modulated by differences in language experience (Figure 7). Recall here that the HSs and L2 learners showed differing patterns: the L2 learners showed a positive correlation between alpha power and RTs for inhibitory control, whereas the HSs showed a negative correlation between alpha power and task performance for this contrast.

The asymmetry in effects provides some support for the predictions of the UBET framework (DeLuca et al., 2020). Specifically, the decreased alpha power seen within the HS group can be interpreted as a measure of increased efficiency. Recall that the EEG data included within the model (see Section **Interaction brain and behavioral results**) came from a *positive* alpha cluster; that is, an increase in alpha power for incongruent relative to congruent trials. As increases in alpha per condition can be interpreted as inhibition of task-irrelevant information (see e.g., Van Diepen et al., 2019), the negative correlation here indicates that the HSs seem to rely *less* on inhibitory control processes to achieve similar reaction time performance for interference suppression.

The pattern seen in the L2 learners was not expected, but also suggests an adaptation to the nature of language control demands. The positive correlation between alpha power and reaction time indicates a shifting adaptation to inhibitory control costs that might not have (yet) reached a degree of efficiency noted in the HSs. This indicates a greater requirement/focus on specific cognitive networks related to language- and domain-general cognitive control. This effort then would lead to a slight slowing on trials requiring increased inhibitory control (such as the incongruent trials), resulting in larger interference effects at the level of RTs.

It is possible that with prolonged (or more intense) engagement with the L2, a broader cognitive control network would be engaged. Recall that the language demographics for the L2 learner group is characterized by L1-dominance (German) with relatively shorter exposure to the non-native language (see Supplementary Table 1). Given the decreased opportunities for engagement and the shorter time frame overall to engage in language control, this could reflect a system that is not fully optimized to handle the control demands (Grundy et al., 2017; Pliatsikas, 2020). It is possible that with increased intensity of use or prolonged duration of use, the patterns seen in this group would more closely align with those seen in the HS group, but further research targeting individuals and groups with such demographic patterns is required to assess this.

## Conclusion

Investigating EEG oscillations in a Flanker task, this study examined potential neurocognitive adaptations in two types of bilinguals. Motivated by inherent differences in duration (and entailed intensity by contextual distinctions) of bilingual experience, we hypothesized that our groups would diverge relative to each other: L2 learners would exhibit increased theta activation while HSs would show increased alpha suppression. However, between-group comparisons revealed no such distinctions. In line with the literature seeking to reveal—if existent in a given aggregate—and understand individual differences in bilingual language and executive control, we regressed various non-neural (e.g., demographic) variables to probe for prediction of individual differences in neural activation. When examining the Flanker effect separately within each group, we did find that both groups demonstrated (partial) signatures of cognitive control (specifically theta increase, initial alpha increase followed by later alpha decrease as well as low beta decrease). Indeed, the data revealed specific bilingual experiential factors correlated to modulatory effects in each group, but differentially so. Moreover, the two groups demonstrated distinct relationships between oscillatory dynamics and behavioral performance on the Flanker task (reaction times). Overall, insights from this study support the view that individual engagement matters in bilingualism and neurocognitive outcomes, but not necessarily in the same way across all bilingual(type)s. That said, it is also prudent to point out that our study is limited to a single cognitive task. That is, to the extent that Flanker is a good task to examine inhibitory control, the present can speak only to support our general conclusions for the relevant cognitive domain. And yet, claims of bilingualism—whether treated/envisaged as a continuous variable or not—potentially affecting executive functions are not limited to the domain of inhibitory control. And so, a modicum of caution needs to be applied in terms of the generalizability of even what the present data support: while we maintain the present study shows that degree of bilingual engagement matters—differentially so for distinct types of bilinguals—for neurocognitive outcomes, this does not mean it affords supportive evidence beyond the tested domain.

## Data availability statement

The original contributions presented in the study are included in the article/Supplementary material, further inquiries can be directed to the corresponding author/s.

## Ethics statement

This study involved human participants. It was reviewed and approved by the University of Konstanz Ethical Committee. The participants provided their written informed consent to participate in this study.

## Author contributions

The present study formed part of a doctoral thesis completed by SMPS. SMPS contributed to the design and conducted the experiments. SMPS, YP, and VD performed the data-analyses. SMPS, YP, VD, and JR contributed to the writing of the manuscript. All authors contributed to the article and approved the submitted version.

## Funding

This article was supported by generous funding to SMPS by the European Union's Horizon 2020 research and innovation programme under the Marie Skłodowska Curie Grant Agreement No. 765556. JR, YP, and VD were funded by the AcqVA Auora Center grant. JR and YP received funding from the Tromsø Forskningsstiftelse (Tromsø Research Foundation) Grant No. A43484 and the Heritage-bilingual Linguistic Proficiency in their Native Grammar (HeLPiNG) (2019–2023).

## Conflict of interest

The authors declare that the research was conducted in the absence of any commercial or financial relationships that could be construed as a potential conflict of interest.

## Publisher's note

All claims expressed in this article are solely those of the authors and do not necessarily represent those of their affiliated organizations, or those of the publisher, the editors and the reviewers. Any product that may be evaluated in this article, or claim that may be made by its manufacturer, is not guaranteed or endorsed by the publisher.

## Footnotes

^1^For 5 participants the low cut-off was adjusted to 1 Hz in order to filter out recurrent skin artifacts.

^2^As argued in the UBET framework (DeLuca et al., 2020), greater intensity of exposure, in isolation, should result in adaptations to increased control demands. However, in combination with prolonged duration of bilingual exposure, increased intensity would *decrease* the latency by which adaptations to efficiency in executive control would occur. Indeed, such trends have been seen in participant cohorts in intensive L2 immersion environments when compared to participants with potentially longer overall duration of use but lower intensity (see for comparison e.g., Burgaleta et al., 2016; Pliatsikas et al., 2017). Given our L2 learners are outside of immersion where English has a clear functional, but not wide societal dispersion, even for individuals who report a young English AoA by virtue of early classroom exposure such a dual language context is not equivalent to the intensity of two languages in a HS context where both languages are used across familial, social, and other contexts. And so, we tested the hypothesis that in our L2 learners compared to HSs this would play out empirically.

## Supplementary material

Supplementary Table 1 Participants' metadata.
